# Reply to Amacher *et al.*

**DOI:** 10.1093/icvts/ivad116

**Published:** 2023-07-20

**Authors:** Zaki Haidari, Matthias Thielmann, Theodor Fischlein, Jurij Matija Kalisnik

**Affiliations:** Department of Thoracic and Cardiovascular Surgery, West German Heart and Vascular Center Essen, University Hospital Essen, Essen, Germany; Department of Thoracic and Cardiovascular Surgery, West German Heart and Vascular Center Essen, University Hospital Essen, Essen, Germany; Department of Cardiac Surgery, Klinikum Nürnberg, Paracelsus Medical University, Nuremberg, Germany; Department of Cardiac Surgery, Klinikum Nürnberg, Paracelsus Medical University, Nuremberg, Germany

**Keywords:** *Staphylococcus aureus*, Infective endocarditis, Surgery, Haemoadsorption, Sepsis

Dear Editor,

We thankfully receive the constructive comments of Amacher *et al.* [1] on our recent publication [2].

In the present analysis, all consecutive patients with confirmed *Staphylococcus aureus* infection were included. The decision to apply intraoperative haemoadsorption was solely at the operating surgeon’s discretion based on unspecified clinical characteristics, favouring in cases with signs of active infection (i.e. fever) and haemodynamic instability (i.e. high vasopressor support). Selection bias was present and this is stated in the limitations section.

The present study differs from our previously published study [3]: in the current analysis, (i) only patients with confirmed *S. aureus* bacteraemia have been selected and (ii) it contains data from 2 centres (Nuremberg and Essen). Our previous publication represents a single-centre evaluation of high-risk infective endocarditis patients irrespective of the underlying bacterial pathogen.

We excluded isolated tricuspid valve endocarditis as this is usually operated on CPB without aortic cross-clamping.

The calculation of number needed to treat is based on absolute risk reduction [4] and the current analysis reported an 18.7% absolute mortality reduction at 90 days which translates to a needed to treat of ∼5, as referenced in the article. We agree that this is a very meaningful clinical benefit, and we believe that this may partly be the result of the simultaneous removal of both cytokines and *S. aureus*-specific toxins in this high-risk population. In comparison to the REMOVE trial [5] and the study published by Santer *et al.* [6], it is important to note that the study populations comprised unselected infective endocarditis patients undergoing surgery. Specifically, patients with *S. aureus* infective endocarditis ranged from 24% to 34%. Therefore, the population in the current analysis is distinctly different and not comparable to previous studies.

The conclusion part of the article states modestly: ‘We could show that intraoperative hemoadsorption appears to attenuate the severity of postoperative sepsis, reducing not only the need for vasopressors, but also 30- and 90-day mortality in patients undergoing cardiac surgery for infective endocarditis caused by *Staphylococcus aureus*. Future studies are essential to confirm the current results in a larger population and to investigate the mechanisms underlying these effects.’ This can be indeed implemented in the take-home message of the visual abstract, like: ‘intraoperative haemoadsorption might improve clinical outcomes in patients after surgery for active *Staphylococcus aureus* endocarditis’.

The manufacturer had no role in the design, conduction and analysis of the present study. Professor Wendt was a staff operating surgeon at University Hospital Essen and is an employee of CytoSorbents©, as of January 2022. It is important to clarify that the last patient from University Hospital Essen included in this analysis was operated in November 2021, before Professor Wendt was employed by CytoSorbents. Dr. Zaki Haidari declare retrospectively his conflict of interests as he received speaker and travel honoraria from CytoSorbents, after the submission of this study.
